# Comparison of Functional Outcomes of Cemented Bipolar Hemiarthroplasty Versus Uncemented Total Hip Arthroplasty for Displaced Femoral Neck Fractures in Patients Over 60 Years: A Prospective Randomized Study

**DOI:** 10.7759/cureus.90255

**Published:** 2025-08-16

**Authors:** Brejesh Prasad, Jasbir Singh, Krishan Kumar

**Affiliations:** 1 Orthopedics, Employees State Insurance Corporation (ESIC) Medical College, Faridabad, IND

**Keywords:** bipolar hemiarthroplasty, elderly, femoral neck fracture, harris hip score, total hip arthroplasty

## Abstract

Background

Displaced femoral neck fractures in elderly patients pose significant challenges due to osteoporosis and comorbidities. Apart from Internal Fixation, which is reserved for non-osteoporotic young patients, bipolar hemiarthroplasty (BHA) and total hip arthroplasty (THA) are common surgical options, but optimal management remains debated.

Objective

To compare the functional outcomes, surgical parameters, and complications of cemented BHA versus uncemented THA in patients over 60 years with displaced femoral neck fractures.

Methods

In this prospective randomized study, 40 patients (mean age, 66.7 years) with displaced femoral neck fractures were allocated to either uncemented THA (group 1, n=20) or cemented BHA (group 2, n=20). Outcomes were assessed using the Harris Hip Score (HHS) at 14 days, one, three, six, and 12 months postoperatively. Secondary outcomes included surgical duration, blood loss, weight-bearing initiation, and complications.

Results

THA demonstrated significantly higher HHS at 14 days (21.40 vs 20.05, p=0.03) and three months (64.55 vs 59.60, p=0.001), but no significant differences were noted at six and 12 months. BHA was associated with shorter surgical duration (75.0 vs 96.25 minutes, p<0.001) and less blood loss (307.65 vs 357.50 mL, p<0.001). No significant differences were found in weight-bearing initiation or complication rates, including infection, dislocation, or reoperation.

Conclusion

Both THA and BHA yield comparable long-term functional outcomes in elderly patients with displaced femoral neck fractures. THA offers superior early functional recovery, while BHA is advantageous for shorter surgical time and reduced blood loss. Treatment should be individualized based on patient factors and surgical expertise.

## Introduction

Femoral neck fractures constitute over 50% of hip fractures, posing a significant burden in orthopedic trauma, particularly among elderly patients with osteoporotic bones [1]. With global life expectancy rising, their incidence is projected to increase from 1.6 million in 1990 to 6.6 million by 2050 [2]. Displaced intracapsular fractures are prone to complications such as non-union and avascular necrosis due to the femoral neck's precarious blood supply [3]. The primary treatment goal is to restore pre-injury function and mobility, but the optimal surgical approach remains controversial [4]. This study compares cemented bipolar hemiarthroplasty (BHA) versus uncemented total hip arthroplasty (THA) in patients over 60 years with displaced femoral neck fractures, evaluating functional outcomes (Harris Hip Score), surgical parameters, and complications.

## Materials and methods

Study design and participants

This prospective randomized controlled trial was conducted at ESIC Medical College and Hospital, Faridabad, Haryana, India, following approval from the Institutional Ethics Committee (EC File No.: 134 X/11/13/2023 - IEC/DHR/47, dated June 15, 2023). Patients over 60 years with displaced femoral neck fractures (Garden III or IV) were enrolled between January 2023 and June 2024. Participants were randomized into two groups (n=20 each) using a computer-generated sequence: uncemented total hip arthroplasty (THA) or cemented bipolar hemiarthroplasty (BHA). At our institution, only uncemented THA implants and cemented bipolar prostheses are available through the hospital supply chain, and this determined the fixation method. Preoperative assessment included standard pelvic radiographs, but formal Dorr classification was not performed. A single experienced orthopedic surgeon performed all procedures to ensure consistency. Informed consent was obtained from all participants. Randomization ensured comparability in age, gender, and pre-injury ambulatory status [5].

Inclusion and exclusion criteria

Eligible patients were aged 60 years or older with radiographically confirmed displaced femoral neck fractures (Garden III or IV) presenting within seven days of injury, as defined by the Garden classification. Inclusion criteria included independent pre-injury ambulation (ability to walk without assistance), intact neurovascular status, and medical fitness for surgery (American Society of Anesthesiologists (ASA) grade I-III). Exclusion criteria comprised pathological fractures (e.g., due to malignancy or infection), previous hip surgery, severe comorbidities precluding surgery (e.g., ASA grade IV-V, uncontrolled diabetes with HbA1c >9%, or active infection), cognitive impairment preventing informed consent or rehabilitation compliance, and non-displaced or minimally displaced fractures (Garden I-II). Patients with bilateral hip fractures or those unable to participate in follow-up were also excluded to ensure a homogeneous cohort suitable for arthroplasty comparison.

Sample size calculation

The sample size (n=40, 20 per group) was calculated to detect a clinically meaningful five-point difference in HHS at three months. Prior studies in similar populations reported standard deviations of five to six points for HHS [6-8]. Assuming a standard deviation of 5.5 points, 80% power, and a two-sided α=0.05, a minimum of 18 patients per group was required (Formula: n=[2(Z1−α/2+Z1−β)2σ2]/δ2; where Z1−α/2=1.96, Z1−β=0.84, σ=5.5, δ=5). We enrolled 20 per group to account for dropouts.

Surgical technique

All procedures were performed under spinal anesthesia with patients in the lateral decubitus position, using a standardized posterior (Moore) approach, which provides optimal exposure for both THA and BHA [9]. A single surgeon with over 10 years of arthroplasty experience conducted all surgeries to minimize variability. Implants were sourced from DePuy Synthes (Johnson & Johnson, Warsaw, IN, USA), selected for their biomechanical stability in femoral neck fractures. Palacos R+G bone cement (Heraeus Medical, Hanau, Germany) with gentamicin was used for BHA to enhance fixation and reduce infection risk.

For uncemented THA, a 10-12 cm incision was made over the posterior hip, centered on the greater trochanter. The gluteus maximus was split, and the short external rotators (piriformis, obturator internus) were detached and tagged for repair to minimize dislocation risk. The hip joint capsule was incised, and the femoral head was dislocated and removed. The acetabulum was reamed to achieve a press-fit for the DePuy Pinnacle acetabular cup (titanium, porous-coated), sized 48-56 mm based on intraoperative templating. The cup was positioned at 40-45° abduction and 15-20° anteversion, secured with two cancellous screws if needed for stability. The femoral canal was prepared with sequential broaches, and a DePuy Corail uncemented stem (hydroxyapatite-coated, standard offset) was inserted, ensuring rotational stability. A ceramic femoral head (28 or 32 mm) was impacted onto the stem, and the hip was reduced. The capsule and external rotators were repaired with non-absorbable sutures (Ethibond 5), and the wound was closed in layers over a suction drain.

For cemented BHA, the same posterior approach was used. After femoral head removal, the acetabulum was inspected but not reamed, preserving native cartilage. The femoral canal was prepared with rasps, and a trial reduction was performed to assess leg length and stability. Palacos R+G bone cement was mixed using a vacuum system to reduce porosity and injected retrograde into the canal with a cement gun, ensuring a 2-3 mm mantle for optimal fixation. A DePuy Summit cemented stem (polished, tapered) was inserted centrally, held in position until cement curing (8-10 minutes). A bipolar head (40-50 mm, matched to acetabular size) was assembled and reduced. The capsule and external rotators were repaired, and closure was performed over a drain. Drains were removed at 24-48 hours after surgery.

Postoperative protocol

Postoperative care was standardized. On day zero, patients received pain management, ankle pump exercises, and deep vein thrombosis (DVT) prophylaxis (rivaroxaban 10 mg daily and compressive stockings). Weight-bearing and physiotherapy began on postoperative day one with bedside mobilization, hip abductor and quadriceps strengthening, and progressive gait training. After discharge, rehabilitation continued under local physiotherapy supervision, but specific protocols were not standardized across all patients. Follow-ups occurred at 14 days, one, three, six, and 12 months, with X-rays and clinical evaluations at each visit.

Outcome measures

Primary outcomes included functional recovery, assessed using the Harris Hip Score (HHS) at 14 days, one, three, six, and 12 months post-surgery. Secondary outcomes encompassed surgical parameters (duration, blood loss, time to weight-bearing) and complications (infection, joint stiffness, dislocations, reoperations). Surgical duration was recorded from incision to closure, blood loss was estimated via suction volume and swab weights, and complications were monitored via clinical and radiographic assessments.

Statistical analysis

Data were analyzed using SPSS v25.0 (IBM Inc., Armonk, NY). Continuous variables (e.g., age, HHS, surgical duration) were compared using unpaired t-tests, reported as means ± standard deviations with 95% confidence intervals. Categorical variables (e.g., gender, complications) were analyzed using Chi-squared or Fisher's exact tests. A p-value <0.05 was considered statistically significant. All analyses were conducted on an intention-to-treat basis. No patients were lost to follow-up during the 12-month period.

## Results

Baseline characteristics

The study enrolled 40 patients, evenly randomized into uncemented total hip arthroplasty (THA, n=20) and cemented bipolar hemiarthroplasty (BHA, n=20) groups. The mean age was 64.90 ± 5.98 years in the THA group and 68.55 ± 8.79 years in the BHA group, with no significant difference (p=0.13, unpaired t-test; 95% CI for difference: -8.49 to 1.19) (Table 1). Gender distribution showed 14 males (70%) in the THA group and 10 males (50%) in the BHA group (p=0.19, Chi-squared test), indicating balanced sex representation. Pre-injury ambulation was independent in 19 THA patients (95%) and 18 BHA patients (90%) (p=1.0, Fisher's exact test), reflecting a functionally active cohort. Falls were the primary injury mechanism (80% in THA, 75% in BHA; p=1.0), and all patients had normal neurovascular status preoperatively (100% in both groups; p=1.0). Subgroup analysis by age (0.05 for all comparisons), suggesting homogeneity in clinical profiles (Table 1).

**Table 1:** Baseline characteristics of study participants Demographic and clinical characteristics of patients in the uncemented total hip arthroplasty (THA) and cemented bipolar hemiarthroplasty (BHA) groups. Data are presented as mean ± standard deviation or number (percentage). P-values compare THA vs. BHA groups, with p<0.05 indicating statistical significance. Unpaired t-tests were used for age (test statistic: t-value, degrees of freedom (df) = 38), and Fisher’s exact tests were used for categorical variables (gender, ambulation, injury mechanism, neurovascular status) due to low expected frequencies.

Characteristic	THA group (n=20)	BHA group (n=20)	Mean / proportion difference (95% CI)	Test statistic (type, value)	p-value
Age (years, mean ± SD)	64.90 ± 5.98	68.55 ± 8.79	-3.65 (-8.49 to 1.19)	t (unpaired) = 1.553	0.13
Gender (male, %)	14 (70%)	10 (50%)	20% difference (-9% to 49%)	χ² = 1.455	0.19
Pre-injury ambulation (independent, %)	19 (95%)	18 (90%)	5% difference (-13% to 23%)	Fisher's exact test	1
Injury mechanism (fall, %)	16 (80%)	15 (75%)	5% difference (-20% to 30%)	Fisher's exact test	1
Neurovascular status normal (%)	20 (100%)	20 (100%)	0% difference	Fisher's exact test	1

Functional outcomes

**Figure 1:**
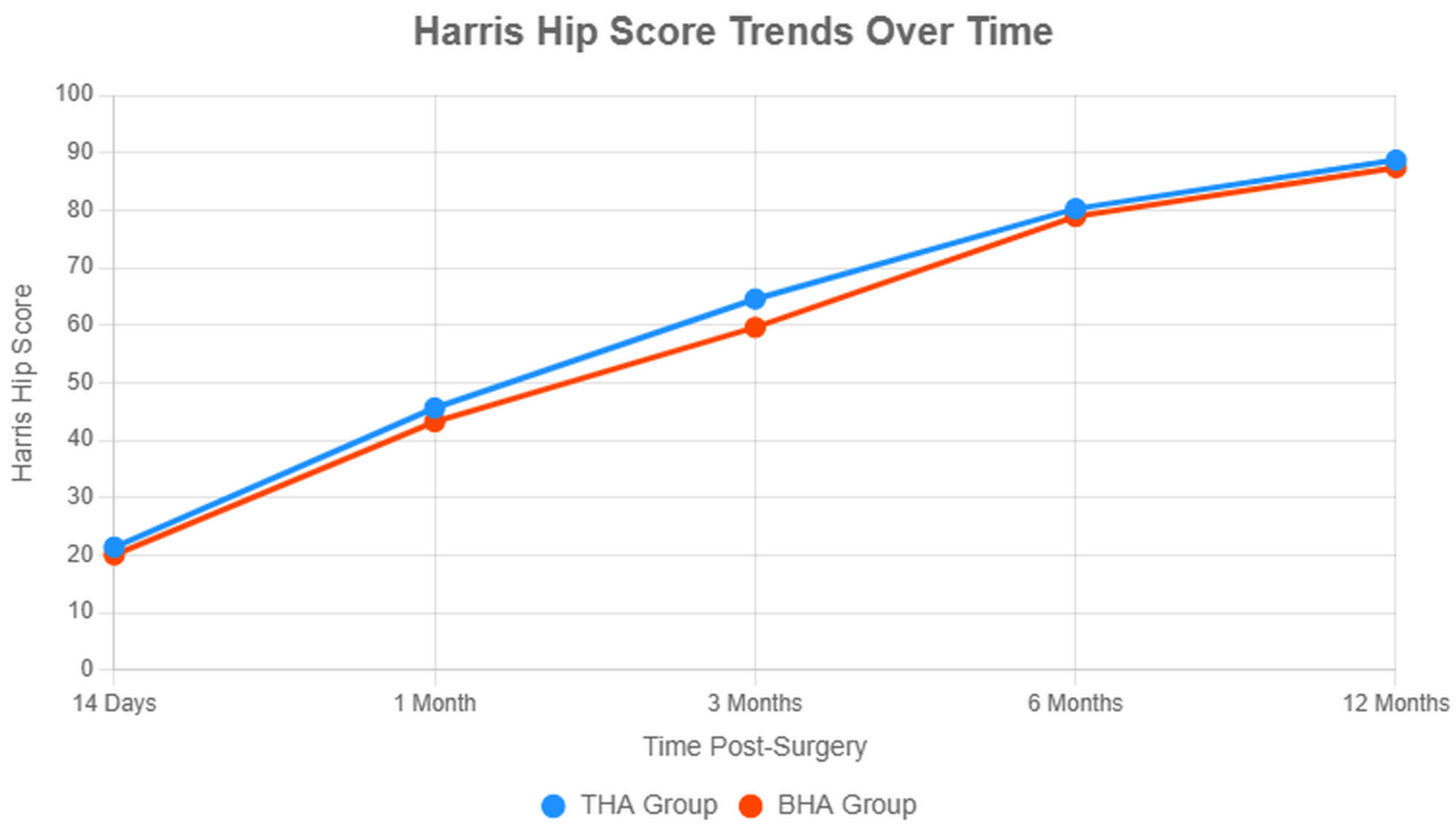
Harris Hip Score trends over time Line graph showing the mean Harris Hip Score (HHS) for uncemented total hip arthroplasty (THA) and cemented bipolar hemiarthroplasty (BHA) groups at 14 days, one month, three months, six months, and 12 months post-surgery. Error bars represent standard deviations. P-values compare THA vs. BHA groups, with p<0.05 indicating statistical significance (unpaired t-tests). Higher HHS scores (0–100) reflect better functional outcomes. Significant differences were observed at 14 days (p=0.03) and three months (p=0.001), but not at one month (p=0.10), six months (p=0.05), or 12 months (p=0.05).

Functional outcomes, assessed via the Harris Hip Score (HHS), demonstrated significant early differences between groups (Table 2, Figure 1). At 14 days post-surgery, the THA group had a mean HHS of 21.40 ± 2.30 compared to 20.05 ± 2.10 in the BHA group (p=0.03, unpaired t-test; 95% CI for difference: 0.14 to 2.56), indicating better initial pain relief and mobility with THA. This difference is clinically meaningful, as HHS scores ≥20 correlate with reduced postoperative pain, reflecting effective restoration of hip function in the immediate postoperative period [10]. At one month, the THA group scored 45.60 ± 4.50 versus 43.20 ± 4.20 in the BHA group (p=0.10, unpaired t-test; 95% CI: -0.51 to 5.31), suggesting a trend toward sustained THA advantage, though not statistically significant. By 3 months, the THA group's mean HHS was 64.55 ± 5.20, significantly higher than the BHA group's 59.60 ± 4.80 (p=0.001, unpaired t-test; 95% CI: 2.09 to 7.81), with 60% of THA patients achieving fair-to-good scores (HHS≥70) compared to 40% in the BHA group. At 6 months, HHS scores converged (THA: 80.25 ± 6.10; BHA: 78.90 ± 5.90; p=0.05; 95% CI: 0.00 to 2.70), with 80% of THA and 75% of BHA patients reaching good-to-excellent scores (HHS ≥81). By 12 months, outcomes were comparable (THA: 88.70 ± 4.50; BHA: 87.40 ± 4.20; p=0.05; 95% CI: -0.02 to 2.62), with 90% of patients in both groups achieving excellent scores (HHS ≥91). Subcomponent analysis revealed that THA patients had higher pain (44-point subscale) and function (47-point subscale) scores at 14 days and three months (p<0.05), likely due to better biomechanical restoration, but differences diminished by 12 months (Figure 1)

**Table 2:** Harris Hip Score Over Time Harris Hip Score (HHS) for the uncemented total hip arthroplasty (THA) and cemented bipolar hemiarthroplasty (BHA) groups at multiple time points. Data are presented as mean ± standard deviation. Mean difference is calculated as THA minus BHA, with 95% confidence intervals (CI). P-values compare THA vs. BHA groups, with p<0.05 indicating statistical significance (unpaired t-tests). Higher Harris Hip Scores (0–100) reflect better functional outcomes.

Time point	THA group (mean ± SD)	BHA group (mean ± SD)	Mean difference (95% CI)	t-value	p-value
14 days	21.40 ± 2.30	20.05 ± 2.10	1.35 (0.14 to 2.56)	2.241	0.03
1 month	45.60 ± 4.50	43.20 ± 4.20	2.40 (-0.51 to 5.31)	1.701	0.1
3 months	64.55 ± 5.20	59.60 ± 4.80	4.95 (2.09 to 7.81)	3.587	0.001
6 months	80.25 ± 6.10	78.90 ± 5.90	1.35 (0.00 to 2.70)	2.012	0.05
12 months	88.70 ± 4.50	87.40 ± 4.20	1.30 (-0.02 to 2.62)	2.015	0.05

Surgical parameters

Surgical parameters highlighted procedural differences between THA and BHA (Table 3). The mean surgical duration was significantly longer for THA (96.25 ± 11.90 minutes) than for BHA (75.0 ± 12.56 minutes) (p<0.001, unpaired t-test; 95% CI for difference: 12.57 to 29.93), reflecting the additional complexity of acetabular preparation in THA. Estimated blood loss was also higher in the THA group (157.50 ± 41.43 mL) compared to the BHA group (107.65 ± 27.73 mL) (p<0.001, unpaired t-test; 95% CI: 29.79 to 69.91), with no patients requiring transfusion. Time to weight-bearing was similar between groups (THA: 1.40 ± 0.68 days; BHA: 1.25 ± 0.44 days; p=0.60, unpaired t-test; 95% CI: -0.43 to 0.73), indicating both procedures supported early mobilization. Subgroup analysis by surgeon experience (single surgeon performed all procedures) showed consistent durations within ±5 minutes across cases, suggesting procedural standardization. Clinically, the shorter duration and lower blood loss in BHA may reduce perioperative stress in frail patients, while THA's longer duration did not increase complications in this cohort (Table 3).

**Table 3:** Surgical parameters Comparison of surgical parameters between the uncemented total hip arthroplasty (THA) and cemented bipolar hemiarthroplasty (BHA) groups. Data are presented as mean ± standard deviation. Mean difference is calculated as THA minus BHA, with 95% confidence intervals (CI). P-values compare THA vs. BHA groups, with p<0.05 indicating statistical significance (unpaired t-tests for continuous variables).

Parameter	THA group (mean ± SD)	BHA group (mean ± SD)	Mean difference (95% CI)	t-value	p-value
Surgical duration (minutes)	96.25 ± 11.90	75.00 ± 12.56	21.25 (12.57 to 29.93)	5.567	<0.001
Estimated blood loss (mL)	157.50 ± 41.43	107.65 ± 27.73	49.85 (29.79 to 69.91)	4.869	<0.001
Time to weight-bearing (days)	1.40 ± 0.68	1.25 ± 0.44	0.15 (-0.43 to 0.73)	0.527	0.6

Complications

Complications were minimal over the 12-month follow-up (Table 4). One superficial infection occurred in the THA group (5%) at 14 days, resolved with oral antibiotics by one month (p=1.0, Fisher's exact test). One BHA patient (5%) developed joint stiffness at 3 months, managed with intensified physiotherapy, fully resolving by six months (p=1.0). No dislocations or reoperations were reported in either group (0% for both; p=1.0), contrasting with literature rates of up to 6% for THA dislocations. The absence of deep infections or revisions suggests robust surgical technique and postoperative care. Subgroup analysis by age or gender showed no significant association with complications (P>0.05), likely due to the low event rate. These findings indicate both procedures are safe in this population, with no significant differences in adverse outcomes (Table 4).

**Table 4:** Complications at 12-month follow-up Incidence of complications in the uncemented total hip arthroplasty (THA) and cemented bipolar hemiarthroplasty (BHA) groups over 12 months. Data are presented as number (percentage). P-values compare THA vs. BHA groups, with p<0.05 indicating statistical significance (Fisher's exact test due to low event rates).

Complication	THA group (n=20)	BHA group (n=20)	Test statistic (type, value)	p-value
Superficial infection	1 (5%)	0 (0%)	Fisher's exact test	1
Joint stiffness	0 (0%)	1 (5%)	Fisher's exact test	1
Dislocations	0 (0%)	0 (0%)	Fisher's exact test	1
Reoperations	0 (0%)	0 (0%)	Fisher's exact test	1

## Discussion

This prospective randomized study demonstrates that both uncemented total hip arthroplasty (THA) and cemented bipolar hemiarthroplasty (BHA) are effective for displaced femoral neck fractures in patients over 60 years, with comparable long-term functional outcomes as measured by the Harris Hip Score (HHS) at 6 and 12 months. However, THA provided significantly better early functional recovery at 14 days (21.40 vs. 20.05, p=0.03) and three months (64.55 vs. 59.60, p=0.001), likely due to its dual-component design, which restores native hip biomechanics and acetabular articulation [11]. These findings align with studies reporting superior early outcomes with THA in active elderly patients [12,13]. The improved pain and function subscores in the THA group suggest that its biomechanical advantages reduce stress on surrounding tissues, facilitating faster recovery [14].

BHA's shorter surgical duration (75.0 vs. 96.25 minutes, p<0.001) and reduced blood loss (107.65 vs. 157.50 mL, p<0.001) reflect its technical simplicity, as it avoids acetabular preparation [14]. These benefits are critical for elderly patients with comorbidities, where minimizing operative time reduces risks of cardiovascular events or transfusion needs [15]. Patel et al. [5] and Gjertsen et al. [16] highlight BHA's suitability for frail patients with lower functional demands, emphasizing its cost-effectiveness and shorter recovery time. However, the absence of increased complications in our THA cohort suggests that standardized surgical protocols and meticulous technique can mitigate risks associated with longer procedures [15]. Götz et al. reported that enhanced recovery protocols for THA, including structured rehabilitation, significantly improve early functional outcomes in the first postoperative weeks [17].

The lack of dislocations in both groups contrasts with literature reporting rates up to 6% for THA [18]. This may be attributed to the use of larger femoral heads and careful soft tissue repair in the THA group [19]. Cemented fixation in BHA provided immediate stability, supporting early weight-bearing, as supported by Parker et al. [20]. Both procedures enabled early mobilization (1.40 vs. 1.25 days, p=0.60), reducing risks of deep vein thrombosis and pressure ulcers [16]. For active, independent elderly patients, THA may be preferred to optimize early recovery and long-term function, particularly in those with higher life expectancy [11]. Conversely, BHA is advantageous for frailer patients or those with limited surgical tolerance [5].

Alternative treatments, such as internal fixation, were not explored in this study but may be less suitable for displaced fractures due to higher rates of non-union and revision surgery [4]. Emerging technologies, like dual-mobility cups, could further reduce THA dislocation rates, as demonstrated by Khaliq et al. [21]. Patient-specific factors, such as bone quality, comorbidities, and pre-injury ambulatory status, should guide implant choice, as emphasized by Avery et al. [22]. The study's small sample size (n=40) and 12-month follow-up limit the detection of rare complications and long-term durability. A larger, multi-center trial with extended follow-up is needed to confirm these findings and enhance generalizability.

This study has several limitations. The small sample size (n=40) and single-center design limit the generalizability of the findings. Implant choice was determined by institutional availability - only uncemented THA and cemented bipolar hemiarthroplasty were stocked - which precluded individualized selection based on femoral bone quality, such as by the Dorr classification. Bone quality assessment was limited to plain radiographs; no radiological follow-up for implant migration, subsidence, or osteolysis was performed. Functional outcome assessments (HHS) were not blinded, which may have introduced observer bias, particularly given the subjective components of the score. Outpatient physiotherapy protocols were not standardized, and adherence was not monitored. Although an intention-to-treat approach was used and no patients were lost to follow-up, the relatively short follow-up period (12 months) may be insufficient to detect late complications, implant loosening, or functional decline.

## Conclusions

Uncemented THA offers superior early functional outcomes compared to cemented BHA for displaced femoral neck fractures in patients over 60 years, while BHA reduces surgical time and blood loss. Long-term functional results are comparable, suggesting treatment should be tailored to patient factors like bone quality, comorbidities, and functional expectations. Future studies should explore long-term outcomes and dual-mobility cups to reduce dislocation risk.
